# Factors related to difficult self‐expandable metallic stent placement for malignant colonic obstruction: A post‐hoc analysis of a multicenter study across Japan

**DOI:** 10.1111/den.13260

**Published:** 2018-09-03

**Authors:** Toshio Kuwai, Toshiki Yamaguchi, Hiroki Imagawa, Shuntaro Yoshida, Hiroyuki Isayama, Takeaki Matsuzawa, Tomonori Yamada, Shuji Saito, Mamoru Shimada, Nobuto Hirata, Takashi Sasaki, Koichi Koizumi, Iruru Maetani, Yoshihisa Saida

**Affiliations:** ^1^ Department of Gastroenterology National Hospital Organization Kure Medical Center and Chugoku Cancer Center Kure Japan; ^2^ Department of Endoscopy and Endoscopic Surgery Graduate School of Medicine The University of Tokyo Tokyo Japan; ^3^ Department of Gastroenterology Graduate School of Medicine Juntendo University Tokyo Japan; ^4^ Department of Gastroenterology Cancer Institute Hospital of Japanese Foundation of Cancer Research Tokyo Japan; ^5^ Department of Gastroenterology Tokyo Metropolitan Cancer and Infectious Disease Center Komagome Hospital Tokyo Japan; ^6^ Division of Gastroenterology and Hepatology Department of Internal Medicine Toho University, Ohashi Medical Center Tokyo Japan; ^7^ Department of Surgery, Toho University Ohashi Medical Center Tokyo Japan; ^8^ Department of Digestive and General Surgery Uonuma Institute of Community Medicine Niigata University Medical and Dental Hospital Niigata Japan; ^9^ Department of Gastroenterology Japanese Red Cross Nagoya Daini Hospital Nagoya Japan; ^10^ Division of Surgery Gastrointestinal Center Yokohama Shin‐Midori General Hospital Yokohama Japan; ^11^ Department of Surgery Toyonaka Midorigaoka Hospital Toyonaka Japan; ^12^ Department of Gastroenterology Kameda Medical Center Kamogawa Japan

**Keywords:** colonoscopy, colorectal cancer, intestinal obstruction, risk factor, self‐expandable metallic stent

## Abstract

**Background and Aim:**

Colorectal cancer patients often present with large bowel obstruction. Elective placement of a self‐expandable metallic stent (SEMS) can relieve obstruction, but can be challenging. Previous studies have compared cases by outcomes only, but the present study investigated successful cases only to identify factors related to prolonged and difficult SEMS placement in patients with malignant colonic obstruction.

**Methods:**

A post‐hoc analysis of a multicenter clinical trial conducted between March 2012 and October 2013 at 46 facilities across Japan (UMIN000007953) was carried out; 511 patients who required SEMS placement for acute colorectal obstruction or symptomatic stricture secondary to malignant neoplasm were enrolled. Technical success rates and procedure times were recorded. Clinical and interventional parameters were investigated for their potential effect on procedure time by univariate and multivariate analyses.

**Results:**

Technical success rate of SEMS placement was 98%. Median procedure time was 30 (range, 4–170) min. In 27% of patients, procedure time exceeded 45 min, indicating technically difficult placement. Multivariate analyses showed significant associations between technically difficult placement and a ColoRectal Obstruction Scoring System (CROSS) score of 0 before SEMS placement (odds ratio [OR], 1.6; *P* < 0.05), tumor site in the right colon (OR, 2.5; *P* < 0.0001), stricture length ≥5 cm (OR, 2.2; *P* < 0.001), peritoneal carcinomatosis (OR, 1.7; *P* < 0.05), and multiple SEMS placement (OR, 8.0; *P* < 0.01).

**Conclusion:**

Clinicians must anticipate technical challenges in cases with peritoneal carcinomatosis, a CROSS score of 0, or expansive strictures; such cases require experienced clinicians to carry out SEMS placement.

## Introduction

Colorectal cancer (CRC) is the most common cancer in Japan^1^ and one of the most common cancers worldwide.^2^ It has been reported that approximately 10% of patients with CRC present with large bowel obstruction.^3^, ^4^, ^5^, ^6^ The conventional treatment for such patients is emergency surgery (colectomy or colostomy), which is associated with poor outcomes and high rates of morbidity and mortality.^7^, ^8^, ^9^ Currently, elective placement of a self‐expandable metallic stent (SEMS) can serve to relieve the obstruction, whether as palliative treatment (PAL) in incurable disease (not amenable to colectomy or colostomy) or as a bridge to surgery (BTS) in patients with potentially resectable CRC. Elective SEMS placement is credited with fewer stent‐related complications, such as perforation, stent migration, and recurrent obstruction, and it results in improved outcomes compared with permanent stoma creation or primary anastomosis.^10^, ^11^, ^12^, ^13^, ^14^, ^15^

In recent years, use of colonic SEMS has been advocated in Japan, with coverage by National Health Insurance beginning in 2012. The Colonic Stent Safe Procedure Research Group, in affiliation with the Japan Gastroenterological Endoscopy Society, was also established to ensure procedural safety and efficacy through mini‐guidelines (brief recommendations) for colonic stent placement. In a recent large, prospective, multicenter study, we demonstrated the feasibility of SEMS placement as PAL or BTS for malignant colorectal obstruction.^14^, ^16^ Further analysis of this dataset showed that SEMS placement is safe and effective in patients with acute malignant colonic obstruction, similar to stoma creation in terms of outcomes and complication rates.^14^, ^16^ Another pooled analysis of patients (*n* = 426) from two prospective, multicenter trials of SEMS placement as BTS for malignant colonic obstruction is pending publication. Technical and clinical success rates of two types of stent were 98.1% and 93.8%, respectively, with an 8.5% rate of SEMS‐related complications.

Despite the safety and efficacy amply documented above,^14^, ^16^ adoption of SEMS placement for malignant colorectal obstruction has been slow, primarily as a result of concerns over lengthy and technically difficult procedures in such complex patients.^17^, ^18^ Longer operating time could generally be a predictor of morbidity under such emergency circumstances.^19^ The prolonged procedure time required to insert and position the stent under challenging circumstances not only adds to the patient's surgical burden, but it also increases the risk of intraoperative incidents. Indeed, a large‐scale, prospective, observational study from our group reported severe incidents as a result of the procedure, such as cardiopulmonary arrest during the procedure, and sepsis.^14^, ^16^ Moreover, air insufflation during the procedure is considered a risk factor for bowel perforation.^20^ Therefore, given these facts, we thought that it is very important to shorten the procedure time and identify the risk factors for such difficult cases. However, previous studies have only compared cases based on outcome.

In the present study, a post‐hoc analysis using the dataset from the previous multicenter clinical trial was conducted,^14^, ^16^ and only successful cases were investigated to identify factors that help predict technically difficult SEMS placement in cases of malignant colorectal obstruction by analyzing factors that accounted for prolonged procedure time.

## Methods

### Patient enrolment

A post‐hoc analysis of a prospective, observational, multicenter clinical trial conducted at 46 facilities (14 academic centers and 32 community hospitals) across Japan between March 2012 and October 2013 was carried out. The clinical trial was registered with the University Hospital Medical Information Network Clinical Trial Registry (UMIN000007953) and has been described in detail in previous reports.^14^, ^16^ Methods of SEMS placement were standardized based on previously published data,^18^, ^21^, ^22^ posting the protocols on a website and disseminating the specifics among participating endoscopists in a prestudy workshop on SEMS placement.^23^ Institutional review boards of participating facilities granted approval prior to study initiation, and informed consent was obtained from all patients agreeing to SEMS placement and clinical data registration. All patients were treated for acute colorectal obstruction and had registered at participating facilities through the study website before or immediately after each procedure.

Patients were managed in accordance with the standard medical practices of each participating facility. Patients undergoing SEMS placement prior to scheduled elective resection of primary tumors were classified as BTS, whereas those without scheduled surgeries were considered PAL.

### Inclusion and exclusion criteria

The registry included patients requiring BTS or PAL decompression for obstructive CRC or extracolonic cancer. Diagnosis was based on abdominal radiography, computed tomography (CT), or colonoscopy. Subjects with a history of prior colonic stent placement, disease‐related complications (enteral ischemia, perforation [suspected or impending], intra‐abdominal abscess or perforation, or severe perineoplastic inflammation), contraindications to endoscopic procedures, or any off‐label use of stents were excluded.

### Stent device and procedure

All procedures involved placement of an uncovered enteral colonic stent (WallFlex colonic stent; Boston Scientific Corp., Natick, MA, USA) with mid‐body and proximal flange diameters of 22 or 27 mm and 25 or 30 mm, respectively, and lengths of 6, 9, or 12 cm. Procedural details were presented in the pre‐introduction publicity announcement and posted on the study website.^23^ Guidewires were used to traverse the strictures, inserting a contrast tube into the proximal lumen to fluoroscopically determine stricture length and establish the number of stents required. Stricture location was tagged intra‐ or extraluminally by endoscopic clips, lipiodol, or radiopaque markers at the discretion of the endoscopist.

### Outcome measures

Procedure times were recorded, considering those beyond the 75th percentile as technically difficult placement. Technical success was defined as accurate SEMS placement, conferring adequate stricture coverage on the first attempt, free of procedure‐related adverse events, such as perforation, re‐obstruction, stent migration, infection/fever, abdominal pain, and tenesmus. Perforation was diagnosed based on clinical, radiological, or intraoperative evidence.

Patients were monitored until hospital discharge. As previously stipulated,^16^ clinical success corresponded with resolution of symptoms and radiological relief of obstruction within 24 h, confirmed by a water‐soluble contrast enema study or radiographic improvement.

### Candidate risk factors (clinical and interventional)

Effects of various clinical and interventional factors were investigated in terms of prolonging procedure time, thus reflecting technically difficult SEMS placement. These included the following: (i) patient parameters, including age, gender, Eastern Cooperative Oncology Group (ECOG) performance status (PS), ColoRectal Obstruction Scoring System (CROSS) score,^14^ and time from diagnosis to SEMS placement; (ii) therapeutic parameters, including treatment intent and history, and use of chemotherapy or radiation therapy; (iii) tumor characteristics, including tumor site and origin, completeness of obstruction (defined as inability to pass flatus, lack of water‐soluble contrast passing proximal to the lesion, or lack of an endoscopically visible lumen),^24^ stricture count and length(s), local or distant spread, and presence or absence of ascites; and (iv) interventional practices, including bowel preparation, length and caliber of first‐placed SEMS, number of SEMS used, digestive tract decompression before SEMS placement, biopsy before SEMS placement, stricture marking, and balloon dilation before SEMS placement.

### Statistical analysis

All computations were carried out using standard software (JMP v13; SAS Institute, Cary, NC, USA), with significance set at *P* < 0.05. Univariate and multivariate logistic regression analyses were undertaken, using stepwise variable selection (patient parameters, 5; therapeutic parameters, 5; tumor characteristics, 10; interventional practices, 11) to identify those associated with technically difficult SEMS placement, expressed as odds ratios (OR) and 95% confidence intervals (CI). Variables reaching a 0.25 level of significance in each step of the stepwise procedure were included in the multivariate logistic regression analysis. To exclude the effect of multicollinearity, if the correlation coefficient between pairs of covariates was greater than 0.4, one of the pair of covariates was excluded from the multivariate analysis.

## Results

**Figure 1:**
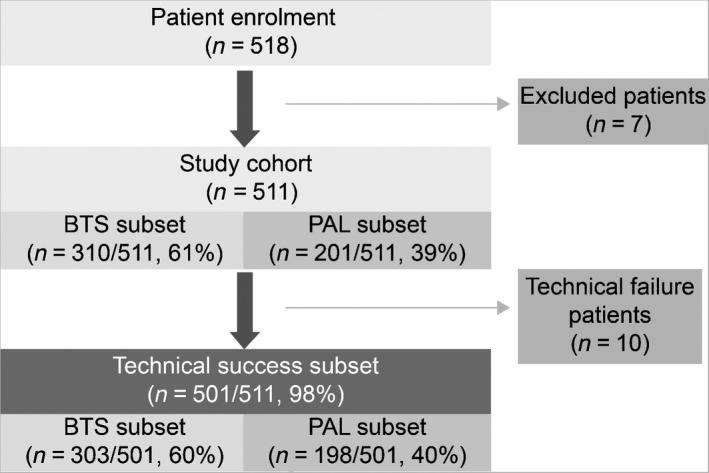
Schematic of the study design and outcomes. BTS, bridge to surgery; PAL, palliative.

A schematic of the study design and results is presented in Figure 1. Although 518 consecutive patients were enrolled, seven failing to meet the study criteria (loose stricture in 3, benign stricture in 3, and nonconforming SEMS device in 1) were excluded. The remaining 511 patients were stratified by treatment intent (per protocol) as BTS (310/511, 60.7%) or PAL (201/511, 39.3%). There were no patient dropouts during the 7‐day follow up, but 10 technical failures occurred because of inability to pass a guidewire through the tumor stricture (*n* = 5), perforation by the guidewire (*n* = 4), and inability to endoscopically visualize the tumor (*n* = 1), resulting in a technical success rate of 98.0% (Figure 1).

### Baseline characteristics

Baseline patient demographic and tumor characteristics in the technical success subset (*n* = 501) are presented in Table 1. Patients’ average age was 70.6 years, and 56.3% of patients were men. PS and CROSS scores were 0 or 1 in the majority of patients. Tumors were commonly located in the left colon (72.7%), and 87.2% of tumors were primary CRC. Clinical success was achieved in 97.6% of patients.

**Table 1:** Baseline patient demographic and tumor characteristics in the technical success subset (*n* = 501)

Characteristic	Value
Age, mean ± SD, y	71 ± 12.8
Gender	
Male	282 (56.3)
Female	219 (43.7)
Performance status	
0/1	349 (69.7)
2–4	152 (30.3)
CROSS score	
0	178 (35.5)
1	148 (29.5)
2	70 (14.0)
3	75 (15.0)
4	30 (6.0)
Tumor site	
Left colon	364 (72.7)
Right colon	137 (27.3)
Tumor origin	
Primary colorectal cancer	437 (87.2)
Locally recurrent colorectal cancer	9 (1.8)
Other extrinsic origin	55 (11.0)
Clinical success	489/501 (97.6)

Data are presented as *n* (%) unless otherwise noted.

CROSS, ColoRectal Obstruction Scoring System.

### Procedure times

**Figure 2:**
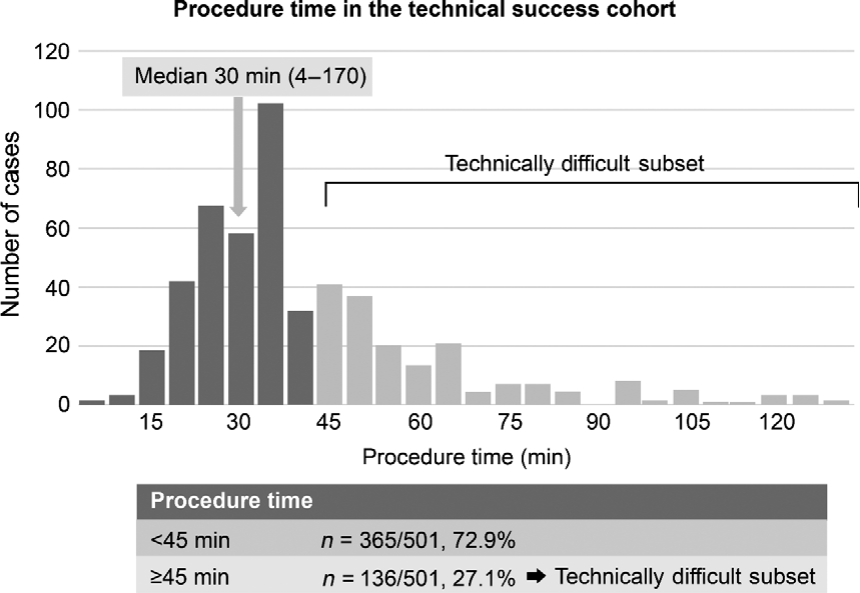
Distribution of procedure times in the technical success subset.

Median procedure time was 30 min (range, 4–170 min). Given that procedure times beyond the 75th percentile qualified as technically difficult placement, 27.1% of procedures were deemed technically difficult, requiring ≥45 min to complete (Figure 2).

### Univariate analysis

Results of univariate analysis are presented in Tables 2, 3, 4, 5. In terms of patient parameters, a CROSS score of 0 and technically difficult SEMS placement trended strongly toward a significant association (OR, 1.5; *P* = 0.07) (Table 2). However, no significant associations were evident between therapeutic parameters and technically difficult SEMS placement (Table 3). In contrast, significant relationships did emerge between technically difficult SEMS placement and tumor characteristics, including tumor site in the right colon (OR, 2.6; *P* < 0.001), multifocal strictures (OR, 11.0; *P* < 0.01), stricture length ≥5 cm (OR, 2.1; *P* < 0.001), and peritoneal carcinomatosis (OR, 2.1; *P* < 0.001) (Table 4). Similarly, significant associations were identified between technically difficult SEMS placement and first‐placed SEMS length >6 cm (OR, 2.6; *P* < 0.0001), placement of multiple SEMS (OR, 6.4; *P* < 0.001), use of a nasointestinal tube (OR, 2.3; *P* < 0.05), and use of a transanal tube (OR, 0.4; *P* < 0.01) (Table 5).

**Table 2:** Univariate analysis of relationships between patient parameters and technically difficult SEMS placement

Parameter	Technical difficulty, *n* (%)	Odds ratio (95% CI)	*P*‐value
Age, y			
<70	58/215 (27.0)	1	
≥70	78/286 (27.3)	1.0 (0.68–1.51)	0.94
Gender			
Male	74/282 (26.2)	1	
Female	62/219 (28.3)	1.1 (0.75–1.65)	0.61
Performance status			
0/1	94/349 (26.9)	1	
2–4	42/152 (27.6)	1.0 (0.68–1.59)	1.04
CROSS score before SEMS placement			
0	57/178 (32.0)	1.5 (0.97–2.20)	0.07
1–4	79/323 (24.5)	1	
Time from diagnosis to SEMS placement, days			
≤3	70/259 (27.0)	1	
>3	66/242 (27.3)	1.0 (0.68–1.50)	0.95

CI, confidence interval; CROSS, ColoRectal Obstruction Scoring System; SEMS, self‐expandable metallic stent.

**Table 3:** Univariate analysis of relationships between therapeutic parameters and technically difficult SEMS placement

Parameter	Technical difficulty, *n* (%)	Odds ratio (95% CI)	*P*‐value
Therapeutic intent			
Bridge to surgery	78/303 (25.7)	1	0.38
Palliation	58/198 (29.3)	1.2 (0.80–1.78)	
Treatment history			
Colon surgery			
Yes	10/48 (20.8)	0.68 (0.33–1.41)	0.30
No	126/453 (27.8)	1	
Other abdominal surgery			
Yes	30/107 (28.0)	1.1 (0.66–1.71)	0.82
No	106/394 (26.9)	1	
Chemotherapy			
Yes	21/71 (29.6)	1.2 (0.66–2.00)	0.62
No	115/430 (26.7)	1	
Radiation			
Yes	1/5 (20.0)	0.7 (0.07–6.03)	0.72
No	135/496 (27.2)	1	

CI, confidence interval; SEMS, self‐expandable metallic stent.

**Table 4:** Univariate analysis of relationships between tumor characteristics and technically difficult SEMS placement

Characteristic	Technical difficulty, *n* (%)	Odds ratio (95% CI)	*P*‐value
Tumor site			
Left colon	79/364 (21.7)	1	<0.001
Right colon	57/137 (41.6)	2.6 (1.69–3.92)	
Complete obstruction			
Yes	118/425 (27.8)	1.2 (0.70–2.19)	0.46
No	18/76 (23.7)	1	
Tumor origin			
Colorectal cancer	115/446 (25.8)	1	0.05
Other	21/55 (38.2)	1.8 (0.99–3.19)	
No. of strictures			
1	132/496 (26.6)	1	<0.01
>1	4/5 (80.0)	11.0 (1.22–99.60)	
Stricture length, cm			
<5	69/320 (21.6)	1	<0.001
≥5	67/181 (37.0)	2.1 (1.43–3.20)	
Tumor with local invasion only			
Yes	65/269 (24.2)	0.7 (0.49–1.07)	0.72
No	71/232 (30.6)	1	
Distant metastasis			
Liver			
Yes	34/135 (25.2)	0.9 (0.56–1.37)	0.55
No	102/366 (27.9)	1	
Lung			
Yes	14/55 (25.5)	0.9 (0.48–1.72)	0.77
No	122/446 (27.4)	1	
Peritoneal carcinomatosis			
Yes	49/127 (38.6)	2.1 (1.35–3.19)	<0.001
No	87/374 (23.3)	1	
Ascites			
Yes	48/156 (30.8)	1.3 (0.86–1.97)	0.22
No	88/345 (25.5)	1	

CI, confidence interval; SEMS, self‐expandable metallic stent.

**Table 5:** Univariate analysis of relationships between interventional practices and technically difficult SEMS placement

Practice	Technical difficulty, *n* (%)	Odds ratio (95% CI)	*P*‐value
Preparation			
Cleansing enema			
Yes	41/172 (23.8)	0.8 (0.50–1.18)	0.23
No	95/329 (28.9)	1	
Oral bowel cleaning			
Yes	8/37 (21.6)	0.7 (0.32–1.62)	0.43
No	128/464 (27.6)	1	
Length of first‐placed SEMS, cm			
6	60/305 (19.7)	1	<0.0001
>6	76/196 (38.8)	2.6 (1.73–3.87)	
Caliber of first‐placed SEMS, mm			
22	128/457 (28.0)	1	0.16
25	8/44 (18.2)	0.6 (0.26–1.26)	
No. of SEMS			
1	127/488 (26.0)	1	<0.001
>1	9/13 (69.2)	6.4 (1.94–21.1)	
Digestive tract decompression before SEMS placement			
Nasogastric tube			
Yes	9/32 (28.1)	1.1 (0.47–2.34)	0.90
No	127/469 (27.1)	1	
Nasointestinal tube			
Yes	18/41 (43.9)	2.3 (1.18–4.35)	<0.05
No	118/460 (25.7)	1	
Transanal tube			
Yes	8/62 (12.9)	0.4 (0.17–0.78)	<0.01
No	128/439 (29.2)	1	
Biopsy before SEMS placement			
Yes	83/292 (28.4)	1 (0.78–1.75)	0.45
No	53/209 (25.4)	1	
Stricture marking			
Yes	76/312 (24.4)	0.7 (0.46–1.03)	0.07
No	60/189 (31.8)	1	
Balloon dilation before SEMS placement			
Yes	2/7 (28.6)	1.1 (0.21–5.61)	0.93
No	134/494 (27.1)	1	

CI, confidence interval; SEMS, self‐expandable metallic stent.

### Multivariate analysis

Results of multivariate analysis (Table 6) aligned with those of univariate analysis, showing significant associations between technically difficult SEMS placement and placement of multiple SEMS (OR, 8.0; *P* < 0.01), CROSS score of 0 before SEMS placement (OR, 1.6; *P* < 0.05), tumor site in the right colon (OR, 2.5; *P* < 0.0001), stricture length ≥5 cm (OR, 2.2; *P* < 0.001), and peritoneal carcinomatosis (OR, 1.7; *P* < 0.05). Multifocal strictures, use of a nasointestinal tube, and biopsy before SEMS placement had no significant relationships with technically difficult placement. Furthermore, significant inverse relationships were observed between technically difficult SEMS placement and digestive tract decompression by a transanal tube before SEMS placement (OR, 0.3; *P* < 0.05), larger caliber (25 mm) of first‐placed SEMS (OR, 0.3; *P* < 0.05), and cleansing enema (OR, 0.5; *P* < 0.01) (Table 6).

**Table 6:** Multivariate analysis of relationships between candidate parameters and technically difficult SEMS placement

Parameter	Technical difficulty, *n* (%)	Odds ratio (95% CI)	*P*‐value
Cleansing enema	41/172 (23.8)	0.5 (0.33–0.86)	<0.01
Larger caliber first‐placed SEMS (25 mm)	8/44 (18.2)	0.3 (0.14–0.82)	<0.05
No. of SEMS placed >1	9/13 (69.2)	8.0 (2.07–31.2)	<0.01
Digestive tract decompression via transanal tube before SEMS placement	8/62 (12.9)	0.3 (0.15–0.78)	<0.05
Biopsy before SEMS placement	83/292 (28.4)	1.4 (0.88–2.19)	0.15
CROSS score of 0 before SEMS placement	57/178 (32.0)	1.6 (1.03–2.59)	<0.05
Tumor site in right colon	57/137 (41.6)	2.5 (1.61–4.01)	<0.0001
Stricture length ≥5 cm	67/181 (37.0)	2.2 (1.38–3.37)	<0.001
Peritoneal carcinomatosis	49/127 (38.6)	1.7 (1.06–2.83)	<0.05

CI, confidence interval; CROSS, ColoRectal Obstruction Scoring System; SEMS, self‐expandable metallic stent.

## Discussion

Results of the present study showed that, despite the complexities of malignant colorectal obstruction (as baseline patient demographic and tumor characteristics attest), technical (98%) and clinical (97.6%) success rates of SEMS placement are high. In nearly 27% of patients, however, procedure time exceeded 45 min, thus qualifying as a technically difficult placement. Furthermore, parameters such as a CROSS score of 0 before SEMS placement, peritoneal carcinomatosis, tumor site in the right colon, stricture length ≥5 cm, and placement of multiple SEMS were significantly associated with or predisposed to technically difficult SEMS placement.

The increased technical difficulty of SEMS placement in the presence of the above factors may be explained in several ways. For example, it is apparent that extreme degrees of distention are the key problem in patients with a CROSS score of 0. Such individuals typically present with severe symptoms, even if continuous decompression is applied.^14^, ^16^ Inevitably, distention at the mouth of an obstruction creates an impediment to guidewire insertion, but post‐stenosis breakthrough examination of the mouth also becomes extremely difficult. Superimposed images of blocked intestinal gas, amidst obstructive enterocolitis, readily impede accurate appraisal of the stenotic segment. As the need for intervention is often urgent, assessing the potential for technical difficulty and, hence, the prospect of a prolonged procedure time, is warranted. It is thus advisable that, under challenging circumstances, SEMS placement be conducted early (obviating the need for gastric tube insertion) and by experienced endoscopists. CROSS scores may be derived from patient symptoms, proving especially useful in anticipating difficulties prior to actual stent placement.

Malignant colorectal obstruction in conjunction with peritoneal carcinomatosis is especially notorious for prolonging SEMS placement procedures. The increased mobility of the bowel in such instances encumbers endoscopic insertion and operation. It may also be difficult to access or accurately identify sites of stenosis secondary to tumor invasion from the serosal surface. Nonetheless, the lower morbidity and mortality rates^25^ achieved through endoscopic stenting in such patients make it preferable to surgery. Thus, SEMS placement should be considered in this context, particularly by an expert endoscopist.

Tumors of the right colon are prone to technical difficulty, no doubt as a result of the greater time required for endoscopic access to obstructive lesions. At a deeper insertion depth, endoscopic maneuverability also suffers, perhaps contributing equally to the overall technical difficulty. It should be noted that obstruction occurring despite the high water content and relatively soft consistency of feces in the right colon suggests a severe degree of luminal stenosis,^26^ promising a longer and technically difficult procedure under compromised conditions.

Lengthy and technically demanding procedures are also expected in patients with stenotic segments ≥5 cm. Passage of guidewires and devices through extended obstructions poses technical problems, requiring particular caution. Similarly, patients requiring more than one SEMS for adequate coverage of expansive stenosis are at a clear disadvantage. Endoscopic operability problems in this setting and efforts to locate points of subsequent SEMS placement carry the risk of stent displacement and heighten the overall complexities of such procedures.

Remarkably, SEMS placement was facilitated by transanal insertion of an ileus tube for preoperative colonic lavage and digestive tract decompression. Upon tube removal, a guidewire is more readily advanced, moving past the point of obstruction with greater ease. Still, we are not endorsing this approach as preparation for SEMS placement, considering the added time, effort, and inherent risk involved.^27^

Several studies compared cases based on outcomes and reported the failure factor of the technical failure cases. Yoon *et al*. retrospectively reported that peritoneal carcinomatosis, extrinsic origin, and tumor site in the right colon were associated with technical failure of stent procedures,^28^ consistent with the present findings. According to our previous prospective study, stricture marking only trended toward a negative association with technical failure, but it was not significant (*P* = 0.09).^16^ Moreover, stricture marking and factors related to technically difficult SEMS placement in this study were few correlated (all correlation coefficients <0.2) and they could be considered completely independent. Therefore, in addition to the present findings, clinicians must also pay attention to these things before SEMS placement.

Limitation of the present study is that it was a post‐hoc analysis with a single‐arm design using only one SEMS device exclusively. Meanwhile, this prospective, multicenter investigation involved a record number of patients, with a high rate of technical success. A fair number of procedures (~25%), however, required a longer amount of time to complete as a result of technical difficulty.

In conclusion, before attempting SEMS placement for obstructive CRC, clinicians must anticipate technical challenges that can occur in patients with peritoneal carcinomatosis, a CROSS score of 0, or expansive strictures. The present findings underscore the need for SEMS placement to be carried out by experienced clinicians in cases with anticipated challenges.

## Conflicts of Interest

T.K. has received grants and personal fees from Boston Scientific Japan, Century Medical Inc., and Olympus Medical Systems Corp.; S.Y. has received personal fees from Boston Scientific Japan, Century Medical Inc., and ZEON Co.; H.I. has received donations and personal fees from Boston Scientific Japan, Century Medical Inc., and Taewoong Medical Devices Inc.; T.M., T.Y., and M.S. have received personal fees from Century Medical Inc.; S.S. has received personal fees from Boston Scientific Japan, Century Medical Inc., and COOK Japan; T.S. has received personal fees from Boston Scientific Japan and Century Medical Inc.; K.K. has received personal fees from Century Medical Inc. and Olympus Medical Systems Corp.; I.M. has received personal fees from Boston Scientific Japan and Century Medical Inc.; and Y.S. has received grants and personal fees from Boston Scientific Japan, Century Medical Inc., and Olympus Medical Systems Corp.; T.Y., H.I., and N.H. have no conflicts of interest or financial ties to disclose.
